# Phytochrome and Phytohormones: Working in Tandem for Plant Growth and Development

**DOI:** 10.3389/fpls.2018.01037

**Published:** 2018-07-27

**Authors:** Panagiotis Lymperopoulos, Joseph Msanne, Roel Rabara

**Affiliations:** New Mexico Consortium, Los Alamos, NM, United States

**Keywords:** plant pathogen, abscisic acid, PIFs, seed dormancy, circadian clock

## Abstract

Being sessile organisms, plants need to continually adapt and modulate their rate of growth and development in accordance with the changing environmental conditions, a phenomenon referred to as plasticity. Plasticity in plants is a highly complex process that involves a well-coordinated interaction between different signaling pathways, the spatiotemporal involvement of phytohormones and cues from the environment. Though research studies are being carried out over the years to understand how plants perceive the signals from changing environmental conditions and activate plasticity, such remain a mystery to be resolved. Among all environmental cues, the light seems to be the stand out factor influencing plant growth and development. During the course of evolution, plants have developed well-equipped signaling system that enables regulation of both quantitative and qualitative differences in the amount of perceived light. Light influences essential developmental switches in plants ranging from germination or transition to flowering, photomorphogenesis, as well as switches in response to shade avoidances and architectural changes occurring during phototropism. Abscisic acid (ABA) is controlling seed germination and is regulated by light. Furthermore, circadian clock adds another level of regulation to plant growth by integrating light signals with different hormonal pathways. MYB96 has been identified as a regulator of circadian gating of ABA-mediated responses in plants by binding to the *TIMING OF CAB EXPRESSION 1*(*TOC1*) promoter. This review will present a representative regulatory model, highlight the successes achieved in employing novel strategies to dissect the levels of interaction and provide perspective for future research on phytochrome-phytohormones relationships toward facilitating plant growth, development, and function under abiotic-biotic stresses.

## Introduction

Plants growth and development is controlled by simultaneously identifying and responding to both the external and internal signals. One well-studied example is the hypocotyl elongation of young seedlings. The light inhibition that provokes the hypocotyl elongation is controlled by the red/far-red light receptor phytochromes and phytohormones, including brassinosteroids (BR), auxin, gibberellins (GA), cytokinins (CK), ethylene, and abscisic acid (ABA). Phytochromes and phytohormones interact to control growth. Multiple genetic studies have proposed that light affects directly the cellular level of some phytohormones and the signal transduction of the vast majority of phytohormones also affect photoreceptor signal transduction. Signal transduction involves the transmission and the conversion of extracellular to intracellular signals into cellular responses where light plays the pivotal factor and can regulate plant growth. Phytochromes are the most important sensors in plants, belonging to a gene family of photoreceptors. They are a family of chromoproteins with a linear tetrapyrrole chromophore. They have two photo-interconvertible forms; the Pr and the Pfr. Pr absorbs red light in a wavelength approximately 667 nm and afterward is converted to Pfr. Plants use the phytochromes to grow in difficult times such as shading and moving toward light PHYB is the significant contributor to germination when seeds have missed low-temperature imbibition. From the other hand, PHYD has also a very important role in full germination when seeds were exposed and stressed in high temperatures during imbibition. Abiotic stresses such as salinity, water stress, drought, high light, high temperatures, harshly affect plant growth and can affect negatively the crop production. Protein phosphatases type 2C (PP2Cs) holds the sucrose non-fermenting 1-related protein kinases (SnRKs) via dephosphorylation in an inactive form (Guo et al., 2011). ABA amount is accumulating and binds to the pyrabactin resistance 1/pyr-like/regulatory components of ABA receptors (PYR/PYL/RCARs) making their structures to interact with PP2C under stress conditions. The resulting interaction makes possible the inhibition of the phosphorylation activity of PP2Cs (Park et al., 2009). Light capacities are able to control multiple responses like seed germination, phototropism, circadian rhythms, flowering, and defense. Plants exposed to natural light comprises different wavelength and hence leads to simultaneous activation of different photoreceptors at the same time. Consequently, this will lead to crosstalk between different signaling pathways thereby leading to a point where the functioning of one may be affected by the other. Even though the developmental processes are regulated normally by the activation of one photoreceptor, but there are some instances where some signaling cascades downstream of light perception of one photoreceptor from same or different classes may be coordinately regulated to ensure successful completion of the process. These developmental processes like seed germination, plant leaf architecture, photomorphogenesis, flowering time or fruit quality essentially require a well-orchestrated activation of different photoreceptors and normally contributes to plant yield *per se* (Mawphlang and Kharshiing, 2017).

## Phytochrome and Light Signaling

Light is one among many essential factors that fuel photosynthesis and make plants grow and develop (Liu et al., 2013c). Plants likewise use light as an informational cue for physiological reactions that are expedited by various cellular responses such as gene expression and protein modification to name a few (Li et al., 2011; Liu et al., 2013c; Kong and Okajima, 2016). Some illustrations of such reactions are seed germination, leaf expansion, organelle movement, directional growth, seedling de-etiolation, stem elongation, shade evasion, flowering, and senescence.

As sessile and autotrophic organisms, plants possess various photoreceptors to absorb light enabling plants to monitor spectral composition, intensity (fluence rate), direction and spatiotemporal patterns of light (Sheerin and Hiltbrunner, 2017). For example, three major photoreceptors in *Arabidopsis thaliana.* These are (1) the phytochromes (phys) that primarily respond to red (R) and far-red light (FR); (2) blue light (B) receptors such as phototropins (phots), cryptochromes (crys) and family members of *ZEITLUPE* (*ZTL*); and (3)*ULTRAVIOLET B* (*UV-B*) *RESISTANCE 8* (*UVR8*) which is sensitive to UV-B light (Li et al., 2011; Tilbrook et al., 2013; Jenkins, 2014; Christie et al., 2015; Kong and Okajima, 2016; Liu et al., 2016). These photoreceptors have chromophores that absorb light signals and able to convert them into biochemical signals such as enzyme activations and protein-protein interactions (Kong and Okajima, 2016).

Phytochromes P were first discovered in plants in 1959 as the light receptor that mediates plant growth and development under long wavelength visible lights (Butler et al., 1959; Rockwell et al., 2006). They can detect and processes R and FR light sources from the environment that primarily support the growth and development of plants. They are also able to measure the ratio of R/FR light and evaluate the extent of photosynthetically active light and also trigger a shade-avoidance response when necessary (Rockwell et al., 2006). Phys can convey information about the immediacy of neighboring plants and accessibility of photosynthetic energy (Chen and Chory, 2011). This family of light receptors exists in two thermally stable conformations, in R (Pr) and FR (Pfr) light-absorbing forms at an inactive 660 nm and an active 730 nm, respectively (Rockwell et al., 2006; de Lucas and Prat, 2014). R-light absorbing converts into FR-light absorbing phytochrome in dark-grown tissues when illuminated with red light thereby triggers photomorphogenesis yet reversible with FR light illumination (Rockwell et al., 2006).

At the molecular level, phytochrome regulates transcription by forming a complex with a family of phytochrome interaction factors (PIFs) (Kong and Okajima, 2016). Aside from PIF, phytochrome also regulates positively acting transcription factors like LONG HYPOCOTYL 5 (HY5) to facilitate photomorphogenesis (Shikata et al., 2014). Recently, phytochrome in *Arabidopsis* was found to induce alternative splicing (AS) cascades in parallel with transcriptional cascades to mediate light responses (Shikata et al., 2014).

In *Arabidopsis*, five well-defined phytochromes named as phyA to phyE have distinct functions in plant growth and development (**Table 1**). Both phyA and phyB are present in monocots and dicots and are considered major clades based on a gene duplication aftermath during early progression of seed plants (Sheerin and Hiltbrunner, 2017).

**Table 1:** Photosensory responses and functional characteristics of phytochromes in the physiological growth and development of *Arabidopsis thaliana*.

Phytochromes	Photosensory	Functional characteristics
phyA	Very LF responses	Seed germination under a broad spectrum of light conditions (UV, visible, FR)
	FR-HI responses	Seedling de-etiolation under continuous FR; promote flowering under LD
phyB	LF responses	Seed germination under continuous R
	R-HI responses	Seedling de-etiolation under continuous R
	EOD-FR (R/FR ratio)	Shade avoidance (petiole and internode elongation, flowering)
phyC	R-HI responses	Seedling de-etiolation in CR
phyD	ED-FR (R/FR ratio)	Shade evasion (flowering, elongation of internode and petiole)
phyE	LF responses	Seed germination
	EOD-FR (R/FR ratio)	Shade evasion (flowering, elongation of internode and petiole)

*(Source: Li et al., 2011). R, red light; CR, continuous red light; FR, far-red light; CFR, continuous far-red light; ED, end of the day; HI, high irradiance; LD, long day light condition; LF, low fluence; UV, ultraviolet.*

A major phytochrome regulating de-etiolation reaction in R light, phyB also mediates R/FR reversible responses and is one of the four light-stable phytochromes (Li et al., 2011). The only light-labile phytochrome phyA mainly mediates photomorphogenic reactions in FR light (Li et al., 2011). PhyA induces flowering, and regulates seed germination, seedling photomorphogenesis, and accumulation of anthocyanins as well as suppresses of hypocotyl growth and inhibits strong canopy shade (Sheerin and Hiltbrunner, 2017).

## Characteristics of Phytochrome Action and Structure in the Cell

Phytochromes exist as homodimers, are soluble proteins and have approximately 125 kDa molecular mass of the apoprotein monomer (Li et al., 2011). In the cytosol, manufacture of phytochrome apoproteins occurs, where the apoproteins accumulate autocatalytically through a linear tetrapyrrole chromophore dubbed phytochromobilin (PΦB) that becomes synthesized once sequential enzymatic reactions transpire in the plastid triggered by 5-aminolevulinic acid (Li et al., 2011).

In the PΦB pathway, two definite steps occur: (1) the early steps involve chlorophyll and heme biosynthesis; and (2) the committed step forms biliverdin IX (BV) when heme oxidizes through a ferredoxin-dependent heme oxygenase (HO) (Li et al., 2011). BV has further broken down into 3Z-PΦB via the enzyme PΦB synthase, later 3Z-PΦB and its isomerized form 3E-PΦB operate as phytochrome chromophore’s functional precursors (Li et al., 2011). Afterward, PΦB is carried across into the cytosol to create holo-Phys upon binding to the newly synthesized apo-Phys (Terry, 1997). A thioether linkage connects the chromophore and an invariant cysteine in a well-conserved area in all phytochromes (Li et al., 2011).

## Phytochrome Interacting Factors (PIFs)

In plants, germination and seedling development are linked to multiple environmental stimuli. Light, temperature, circadian clock, and phytohormones including gibberellins and brassinosteroids (BR) regulate plant growth and development upon seedling emergence (Toledo-Ortiz et al., 2010; Liu et al., 2013c). More importantly, light availability after germination, and upon transition through the soil surface, determines the seedling developmental path with photomorphogenesis occurring under light and skotomorphogenesis (etiolation) under dark conditions (Quail, 2002; Chen et al., 2004). Etiolated seedlings, which extend and elongate while searching for light, accumulate pigment precursors in the plastids (etioplasts) including the chlorophyll precursor protochlorophyllide (Pchlide), as well as carotenoid precursors (Park et al., 2002; Rodríguez-Villalón et al., 2009). Upon light irradiation, seedlings undergo a photomorphogenic development with etioplasts differentiating into chloroplasts, accompanied by the production and accumulation of chlorophylls and carotenoid, and the rapid assembly of a functional photosynthetic system (Toledo-Ortiz et al., 2010; Liu et al., 2013c).

## Biological Function of PIF Proteins

Genetic analyses of mutant and overexpression lines have confirmed the role of PIFs in the regulation of various light-mediated processes, including control of stomatal development, repression of germination and photomorphogenic development under dark conditions, and promoting seedling skotomorphogenesis and shade-avoidance (Duek and Fankhauser, 2005; Leivar et al., 2008; Casson et al., 2009; de Lucas and Prat, 2014). Studies have shown the redundant functions of PIF1, PIF3, PIF4, and PIF5 in elongating the hypocotyl in response to red light conditions (Shin et al., 2009). In the dark, *A. thaliana pif* monogenic mutants displayed constitutive photomorphogenic phenotypes, including open cotyledons, short hypocotyls (similar to wild-type), disrupted gravitropism, and accumulation of higher amounts of Pchlide (Leivar et al., 2008; Shin et al., 2009; Park et al., 2012; de Lucas and Prat, 2014). When the mutant seedlings were transferred to light they were severely bleached (Shin et al., 2009). The *pif* quadruple mutant displayed 40% shorter hypocotyls than wild-type plants and showed upregulation of light-inducible genes even under dark conditions (Leivar et al., 2008; Shin et al., 2009; Park et al., 2012). In contrast, overexpression of *PIFs* in *A. thaliana* resulted in skotomorphogenic phenotypes even under normal light conditions (Lorrain et al., 2008; Xie et al., 2017). Mutant seedlings displayed constitutively long hypocotyls, negative gravitropism, unopened cotyledons, and inhibition of chlorophyll biosynthesis (de Lucas and Prat, 2014). In addition, lines overexpressing *PIFs* exhibited a shade-avoidance syndrome, even under high red to far-red light conditions (Xie et al., 2017).

In *A. thaliana*, several genes have been identified as targets for PIF TFs, which specifically bind to the promoters of several genes that are light responsive by using *cis*-acting regulatory elements such as G-boxes and E-boxes (Shin et al., 2007; Rosado et al., 2016). PIF1 and PIF3 suppress chloroplast development and synthesis of chlorophyll and carotenoid under dark conditions, by negatively down-regulating the expression of key photosynthesis and pigment biosynthesis genes (Stephenson et al., 2009; Toledo-Ortiz et al., 2010). Chlorophyll biosynthetic genes, including *GUN5* encoding for the first enzyme in the tetrapyrrole pathway (Mochizuki et al., 2001), are highly upregulated in the *pif3* mutant seedlings (Shin et al., 2009). The expression of photosynthetic genes such as *LHCA1* and *psaE1* is also found to be upregulated in *pif3* mutants (Shin et al., 2009). PIF3 suppresses chlorophyll biosynthetic and photosynthetic genes by recruiting and direct interaction with the histone deacetylase *HDA15* (Liu et al., 2013b), which reduces the histone acetylation levels and decreases gene transcription (Liu et al., 2013b). PIF1 and other PIFs bind directly to the G-box motif in the promoter region of Phytoene Synthase (PSY) during de-etiolation and thereby inhibits its expression and hence the carotenoid biosynthesis (Toledo-Ortiz et al., 2010; Rosado et al., 2016). In addition, PIF1 negatively regulates seed germination, with *pif1* single mutant showing light-independent germination (Oh et al., 2004).

In addition to PIF3, other PIFs such as PIF4 and PIF5 negatively regulate various light responses in plants (Huq and Quail, 2002; Fujimori et al., 2004). PIF4 represses the expression of the chloroplast activity maintainer genes *GLK1* and *GLK2*, by binding to the E-box motifs of their promoter regions (Song et al., 2014). During dark-induced senescence, PIF4 and PIF5 upregulate the expression of genes encoding for chlorophyll degradation enzymes (*SGR1* and *NYC1*), as well as a master senescence transcription factor (*ORE1*), by binding directly to the G-box motifs in upstream regions (Song et al., 2014; Rosado et al., 2016). PIF4 and PIF5 also adversely affects the phytochrome-mediated inhibition of shade avoidance response (Lorrain et al., 2008; Shin et al., 2009), and participate actively in blue light-induced phototropism (de Lucas and Prat, 2014). In simulated shade, accumulation of PIF4 and PIF5 proteins upregulate the expression of several genes encoding for auxin biosynthesis and involved in the stem and petiole elongation growth and leaf expansion (Lorrain et al., 2008; Leivar and Quail, 2011; de Lucas and Prat, 2014). Under these conditions, PIFs repress the expression of several conserved miRNAs genes (*MIR156*), resulting in altered plant architecture (Axtell and Bowman, 2008; Xie et al., 2017). PIF6 is highly expressed during seed development, and loss of function mutation resulted in increased seed dormancy (de Lucas and Prat, 2014). Finally, PIF7 showing higher stability than other PIFs, yet phosphorylated under light (Leivar et al., 2008), modulate auxin levels in the cotyledons (Li et al., 2012) and plays a minor role in hypocotyl elongation (Shin et al., 2009).

## Regulation of PIF Function in Seed Germination

Although light stimulates photosynthate production, it slows down hypocotyl elongation once photoreceptors are activated, especially the blue-light and red-light absorbing photoreceptors called cryptochromes (crys) and phys, respectively (Vandenbussche et al., 2005). Numerous studies have been conducted on photoreceptor functions exhibiting varying dimerization and phytochrome-binding characteristics and recently centered on PIFs that have been shown to regulate distinct photomorphogenesis functions, including seed germination (Castillon et al., 2007; Stewart et al., 2011). For instance, PIF6 was found to regulate seed germination, while PIF 3, PIF4, and PIF5 influence hypocotyl elongation. PIF1 can regulate both seed germination and hypocotyl elongation (Oh et al., 2004; Penfield and Hall, 2009; Piskurewicz et al., 2009). Similarly, adding green light to blue light partially subdued blue light inhibition of hypocotyl elongation (Li et al., 2011). In mutant plants called PIFq mutants, which lack functions of either or combination of PIF1, PIF3, PIF4, and PIF5, even when grown in complete darkness, show phenocopy transcriptional and morphological reactions of light-grown plants (Stewart et al., 2011).

Seed germination is a plant’s first adaptive decision in its development and advances in genetics and molecular physiology have shown factors that control germination (Penfield and King, 2009). In *Arabidopsis*, induced efficient germination of its dormant seeds requires cold treatment (stratification) and light activation of the photochrome system (Oh et al., 2004, 2006, 2007; Penfield et al., 2005; de Lucas and Prat, 2014). When acting synergistically, light and cold temperatures promote germination in seeds, a reaction regulated by SPATULA (SPT) factor which is light-stable (Penfield et al., 2005). Germination in the dark or light is inhibited, respectively, by PIF1 and bHLH factor SPT, a linked non-phy-interacting factor in Subfamily-15 (Leivar and Quail, 2011). To optimize photomorphogenesis in *Arabidopsis* seedlings, PIF1 is controlled by light-mediated degradation via the ubiquitin-26S proteasome pathway (Shen et al., 2005).

PIF3-LIKE5 (PIL5/PIF1/bHLH015) or PIL5 has different effects on gibberellin genes. It blocks key GA3ox1 and GA3ox2, both GA-biosynthetic genes yet incite the expression of GA2ox2, a GA catabolic gene that results in low GA levels maintenance (Oh et al., 2006). PIL5 inhibits seed germination in *Arabidopsis* (Oh et al., 2004). Further, PIF1 stimulates ABA biosynthetic genes but represses an ABA catabolic gene leading to increases in ABA levels without binding to GA and ABA metabolic gene promoters but adhering directly to GA-INTENSIVE (GAI) and REPRESSOR OF GA (RGA) promoters (Oh et al., 2007). In seed germination, GA biosynthesis is associated with nitrate level that is managed by embryonic ABA (Osuna et al., 2015).

In a study comparing microarray and ChIP-chip analyses of PIF1 during seed germination, PIF1 mediated 166 genes by directly binding to their promoters (Oh et al., 2009). These genes are direct target genes regulated by PIF1 that likewise expedite the downstream facets of PIF1 which represses seed germination (Leivar and Monte, 2014). In short, PIF1 does inhibit seed germination by harmonizing hormone signals and adjusting cell wall properties in imbibed seeds and through biosynthesis of both GA and ABA (Oh et al., 2009).

Results of a high-resolution temporal analysis in seed germination study involving sucrose suggest that known photomorphogenetic signaling network can change due to carbon availability (Stewart et al., 2011). Sucrose prolonged the number of days plants underwent rapid hypocotyl elongation resulting in remarkable increases in ultimate seedling height and altered the timing of daily growth maxima exhibiting more plastic diel growth dynamics (Stewart et al., 2011).

## PIFs Involvement in Hormone Signaling

As hormones essentially contribute to the growth and development of plants, plants have various mechanisms that harmonize hormone-driven processes such as modulating hormone synthesis as well as transporting and signaling (Ljung et al., 2015). These processes consisting of numerous hormonal pathways are often light-regulated by to incite plant growth and development (Ljung et al., 2015).

Recent studies have shown interactions of molecular mechanisms affected by light on hormonal pathways. For instance, PIF3 and PIF4 in *Arabidopsis* are identified pathway integrators among light signaling components that influence seedling photomorphogenesis, particularly integrate light and GA signaling (Ljung et al., 2015). PIF3 and PIF4 also have dual regulation representing an integration point for seedling photomorphogenesis occurrence in response to GA and light, as shown in their interactions with DELLA in photomorphogenesis (de Lucas et al., 2008; Feng et al., 2008; Ljung et al., 2015).

As PIFs are known to compete with GA receptors for DELLA binding, they should be considered to stabilize DELLAs in the presence of GA (de Lucas et al., 2008; Feng et al., 2008). Aside from its upstream functions in ABA and GA metabolism, PIFs are essential for the normal induction of hormone-related gene expression linked to the germinating state via environmental cues (Penfield et al., 2005; Oh et al., 2006) and to a small degree, affect GAI and RGA transcript levels (Oh et al., 2007).

## Phytohormones

Environmental factors such as drought, temperature, cold, pathogens, and salinity affect plants growth and development. For many years scientists have studied the role of phytohormones as plant regulators. Plant hormones or phytohormones are known to be small molecules. They are circulated within tissues (cell), through the intercellular space, and through the vascular bundles. All plant physiological responses are influenced by plant hormones, the chemical messengers that include auxins, cytokinins, ABA, and gibberellins. Plant hormones or phytohormones can affect the major aspects of plant life such as flowering, fruit maturation, and senescence. Phytohormones can regulate cellular activities such as cell division (controlled mainly by cytokinin), cell elongation (controlled mainly by auxins) and differentiation, reproduction, and responses to abiotic and biotic stress. Auxins control the differentiation of meristem into vascular tissue and promote leaf development. There are synthetic auxins that have been used as herbicides but an auxin that shows physiological activity is the indole acetic acid (IAA). Auxins are the class of phytohormones responsible for elongation in phototropism and gravitropism. ABA plays a pivotal role in plants by responding and adapting to environmental stress. Cutler et al. (2010) has reported the importance of ABA in root controlled growth under drought conditions. ABA is also famous for its effect in the maintenance of dormancy buds and its role as an inhibitor of germination. It’s the hormone with the ability to reverses the effect other stimulating in growth hormones such as gibberellins and cytokinins. Another very important plant hormone responsible for seed dormancy, responsible for shoot elongation, seed germination, and fruit and flower maturation is the gibberellins. GAs break seed dormancy in exposure to cold or light and allow the seeds to start the process of germination. An exogenous application of GA reveals a hyper elongation phenotype in stems. GA’s have the ability also to interact with other phytohormones in a synergistic or antagonistic way. ABA plays an antagonistic function to GAs actions because induces dormancy in seeds by blocking germination and promotes the synthesis of storage proteins. Therefore, plants can be adapted to temperature climates where it requires a longer period of low temperatures before germination. This mechanism gives a protecting ability to young plants from too early sprouting.

## Plant Defense Response

Besides the involvement of light signaling in photomorphogenesis and photosynthesis, recent evidence have identified light signaling as a determinant factor in plant–pathogen interactions (Ballaré et al., 2012; Erb et al., 2012). *Cryptochrome 2* (*CRY2*) and *Phototropin 2* (*PHOT2*) have been identified as a mediator of plat defense against viral pathogens via interaction with resistance protein (Jeong et al., 2010). In addition, phytochromes and *UVR8* have been also implicated to play a role in defense response (Mazza and Ballaré, 2015; Gommers et al., 2017). The defense response exerted by the photoreceptors are facilitated by regulating the hormone signaling pathways like salicylic acid (SA) and jasmonic acid (JA) (Xie et al., 2011; Moreno and Ballaré, 2014). In the near future, more reports on the integration of light and hormone signaling pathways will help us gain more insights into the mechanism governing how light interacts with hormonal signaling to influence plant agronomic traits.

## ABA-Regulated Stomatal Closure in Pathogen Response

Abscisic acid-mediated closure of stomata for conserving the water is not only a mechanism to protect against osmotic stress but it also serves as a defense mechanism against pathogen attack. A significant overlap has been observed between the signaling pathways of pathogen resistance and abiotic stress tolerance. ABA not only facilitates defense against a pathogen by promoting stomatal closure but it is also involved in hormonal cross-talk (Melotto et al., 2006, 2008; Mosher et al., 2010). Though plants have physicochemical barriers but the pathogens have mechanisms by which they can easily overcome the defense barriers including cell wall thereby leading to successful infection. Studies over the years have identified that the fungal pathogens can enter the plant cell by using cell-wall degrading enzymes or mechanical pressure but on the contrary bacterial pathogens require a passage to enter the cell-like hydathodes, stomata, and lenticels (van Kan, 2006; Melotto et al., 2008; Ton et al., 2009). Pathogen-Associated Molecular Pattern (PAMP) triggered immune responses in plants includes a stomatal closure that restricts pathogen entry into the cell (Melotto et al., 2006). Analysis of *ost1* (open stomata) and *aba3-1* mutants that are either insensitive or ABA-deficient have shown that PAMP failed to initiate stomatal closure (Melotto et al., 2006). These observations thus clearly suggest that PAMP induced immune response is mediated by ABA by inducing the closure of stomata.

## ABA Regulation of Pathogen Response Through Interplay With Other Hormones

Phytohormones like SA, JA and ethylene play a substantial role in defense response against pathogens. SA is related to SAR (systemic acquired resistance) and biotrophic pathogen resistance while JA and ethylene provide resistance against necrotrophic pathogen and induced systemic resistance (ISR) (Lee and Luan, 2012). ABA has been reported to act either in coordination or antagonistically with SA, JA, or ethylene (Anderson et al., 2004; Mosher et al., 2010). Overexpression of ABA biosynthesis genes *NCED2, NCED3*, and *NCED5* led to increased accumulation of ABA but favored the significant growth of bacteria. Similar observations were on analysis of *Arabidopsis aba3-1* mutant that showed resistance against *Pseudomonas syringae* but showed enhanced susceptibility to bacterial pathogens on the application of exogenous ABA (Fan et al., 2009). These observations suggest that SA mediated pathogen response is negatively regulated by ABA. Moreover, ABA is also reported to repress SA-induced expression of genes thereby suppressing SAR (Yasuda et al., 2008). These results collectively suggest that ABA is a negative regulator of SA induced defense response against pathogens. Again ABA is also reported to negatively regulate JA- and ethylene-dependent resistance against pathogens as observed in the analysis of ABA-deficient mutant in *Arabidopsis* that showed strong induction of JA and ethylene marker genes and downregulated upon exogenous ABA treatment (Anderson et al., 2004). Overexpression of mitogen-activated protein kinase (*OsMPK5*) in rice led to increased susceptibility against *Magnaporthe oryzae* and the bacterial pathogen *Burkholderia glumae* due to increased ABA accumulation and simultaneous reduction in levels of ethylene (Xiong and Yang, 2003; Asselbergh et al., 2008).

## ABA Signaling in Response to Abiotic Stress

Abscisic acid-mediated stress response plays a crucial mechanism to provide protection against environmental stresses like salt, drought, or temperature (Sunitha et al., 2017). The identification of ABA receptors and the identification of the core ABA-dependent pathway is the greatest advancement in the recent past. PYR/PYL/RCAR (PYLs) belonging to the family of START domain proteins were identified as ABA receptors by using chemical genetics and protein-protein interaction studies (Park et al., 2009; Cutler et al., 2010). PYLs bind to ABA in the presence of PP2Cs like ABI1, ABI2, HAB1, and PP2CA and hence it can be said that PP2Cs can be considered as cor-receptors (Ma et al., 2009; Santiago et al., 2009). PP2Cs block the activity of kinases like SnRK2.2, 2.3, and SnRK2.6 (*OST1*) in the absence of ABA by dephosphorylating them (Soon et al., 2012). In the presence of ABA, PP2C activity is inhibited by ABA-PYLs that in turn releases the SnRK2s which gets activated by autophosphorylation and then subsequently leads to the induction of downstream genes (Fujii et al., 2009; Park et al., 2009). *ROP11* a GTPase interacts with ABI1 to prevent inhibition by *PYL9* and in turn, *ABI1* and other *PP2C* prevents ABA-induced degradation of *RopGEF1* (GTP exchange factor). Thus, it can be said that the formation of this control loop help prevents any leaky ABA signaling in the absence of stress (Li et al., 2016). Analysis of knockout lines of PYL8 resulted in ABA insensitivity and late recovery of lateral growth from stress-induced inhibition. It has been proven that stress-induced inhibition of lateral growth occurs via the interaction of *PYL8* with *MYB77, MYB44*, and *MYB 73* which in turn induces the transcription of auxin-responsive genes (Zhao et al., 2014). Again, the interaction of PYL6 with MYC2 transcription factor connects the ABA and jasmonate signaling pathways (Aleman et al., 2016). In addition, many bZIP transcription factors like *ABI5* and ABA-responsive element-binding factors (ABFs) are phosphorylated by *SnRK2* kinases to mediated the ABA-mediated stress response (Furihata et al., 2006). The anion channel protein *SLAC1* associated with the plasma membrane are phosphorylated by *SnRK2* resulted in ABA-induced closure of stomata and subsequent water loss due to transpiration (Geiger et al., 2009, 2011). ABA-induced signaling complex of PYL-PP2C-SnRK2 induces a MAPK cascade that in turn controls the induction of several ABA-inducible genes by phosphorylating them (de Zelicourt et al., 2016). Not only ABA-induced *SnRK2s* to activate the TF cascade but it also facilitates the ROS signaling by inducing NADPH oxidase *RbohF* that produces O_2_ which subsequently produces H_2_O_2_ that ultimately leads to the closure of stomata (Zhu, 2016). Induction of ROS production by ABA has reduced in pip2;1 mutant plants suggesting that H_2_O_2_ enters the cells via aquaporin (Grondin et al., 2015). Analysis of mpk9-1/12-1 double mutants in *Arabidopsis* provided the evidence that MPK9 and MPK12 act downstream and positively regulate the ABA-induced stomatal closure (Jammes et al., 2009). Another important component of ABA-induced stomatal closure is *GHR1* (GUARD CELL HYDROGEN PEROXIDE-RESISTANT1) that phosphorylates SLAC1 to initiate the process but it gets inhibited by *ABI2* but not by *ABI1* (Hua et al., 2012). Calcium signaling also regulates ABA-mediated closure of stomata as observed through the analysis of mutants defective in calcium-dependent protein kinases, *CPK5, CPK6, CPK11*, and *CPK23* (Brandt et al., 2015).

## ABA Regulation of the Circadian Clock

Plant circadian regulation of hormones signaling controls many aspects of development, such as growth patterns and seed dormancy. The circadian clock senses the day-length in the four different seasons, allowing plants slowly to adapt in key development transitions (Dodd et al., 2005). In *Arabidopsis thaliana* have been characterized several circadian clock components such as the *TIMING OF CAB-1* (*TOC1*) gene, the *PSEUDO-RESPONSE REGULATOR-7* and -*9* (*PRR7* and *PRR9*), the *CIRCADIAN CLOCK ASSOCIATED 1* (*CCA1*), and the *EARLY FLOWERING 3* (*ELF3*) (Wang and Tobin, 1998). These well-characterized genes have established many transcriptional feedback loops to ensure strong circadian alternation. A core feedback loop includes two distinct MYB transcription factors; the *LATE ELONGATED HYPOCOTYL* (*LHY*) and the *CCA1*, with mainly common functions.

The plant’s hormone ABA expression is effected and controlled by the clock. During drought stress ABA responses becoming evident during the dusk time (Legnaioli et al., 2009). MYB96 is likely a molecular component responsible for the bidirectional regulation between ABA and the clock. Besides the regulation of MYB96 by the circadian clock, the MYB96 protein subsequently influences clock activity possible via TOC1. The circadian network built up from MYB96 is particularly relevant when ABA is applied. In the presence of ABA, MYB96 activation of TOC1 probably suppresses CCA1 expression. When ABA is applied at certain times of day, other than dusk, the ABA-activated MYB96-TOC1 module suppresses CCA1 activity in order to synchronize the circadian oscillation to the environmental signal (Liu et al., 2013a; Habte et al., 2014). Given that ABA-reduction of circadian period largely depends on MYB96, the MYB96 protein acts as a possible regulator mediating the interaction between the circadian clock and ABA signaling. It has also been shown that that expression of the core clock TOC1 gene is induced by the application of ABA, demonstrating the way of action of ABA (Legnaioli et al., 2009).

## Photoreceptors From Inter and Intraclass Affecting Plant Development

Natural light is composed of different wavelengths that will lead to simultaneous activation of several photoreceptors in plants grown under normal conditions. The activation of different photoreceptors (PR) could induce signaling networks that will result in a crosstalk where the activity of one PR may be affected by the other. The developmental processes in plants are normally regulated by a signal photoreceptor but there are reports where it has been observed that upon perceiving the light stimulus it induces genes downstream of one or multiple PRs of the same or different family that participates in coordination to regulate growth and development effectively. These developmental processes include germination of seedlings (Dechaine et al., 2009), architecture of the plant and leaf (Kozuka et al., 2013), photomorphogenesis (Weller et al., 2004), fruit quality and flowering (González et al., 2015; Endo et al., 2016) that eventually results in plant productivity. Analysis of photomorphogenic mutant and transgenic lines of *Arabidopsis*, rice, tomato, and other model species have enabled dissecting the functional redundancy as well as the signaling networks of different photoreceptors involved in plant growth and development. Plants responses to PR mediated signaling network becomes very crucial when plants are subjected to harsh environments. Thus it can be said that an in-depth understanding of the interaction between different photoreceptors will enable the researchers to make use of this information for improving plant growth and productivity.

## Photoreceptors and Phytohormones: Light-Mediated Hormonal Regulation of Plant Growth Responses

### Seed Germination

Light plays an essential role in plant development like germination, the emergence of a seedling from the soil and photomorphogenesis. For establishing the emergent seedlings involves several coordinately regulated photoreceptors. Light-mediated activation of these photoreceptors, in turn, switches on the signaling d cascades that functionally regulate plant growth and development from directing seedling emergence to establishing them as an autotroph. Studies that have been conducted over the past years have shown that these photoreceptors mediated signaling cascade involves the interaction of light with phytohormone mediated signaling pathways (Wang et al., 2013). After the initial theory of Cholodny–Went that hypothesized on the asymmetric distribution of auxin during phototropism many reports have come justifying the involvement of light perception and hormonal control in plant development. GA and ABA are known to play contrasting roles in the developmental processes of plants where one favors germination while the other inhibits it (Jacobsen et al., 2002; Seo et al., 2009). Interaction of phytochromes and its partners *PIF1* or so called *PIL5* facilitates germination upon light activation by regulating ABA and GA signaling through its downstream targets (Oh et al., 2009; Seo et al., 2009; de Wit et al., 2016). Phytochrome-mediated degradation of *PIF1* is proposed to be the crucial mechanism that controls light induced seed germination by altering the metabolism of ABA and GA in seeds. In addition, PIFs have also been associated with the transition of seedlings from skotomorphogenic to a photomorphogenic development mode by altering the levels of GA (Mawphlang and Kharshiing, 2017). Basic leucine zipper (bZIP) transcription factor *Elongated Hypocotyl5* (*HY5*) have been implicated to be the common regulatory node for several regulators of hormone signaling in *Arabidopsis* that includes auxin and cytokinin (Cluis et al., 2004), GA (Alabadí et al., 2008), brassinosteroid (Li and He, 2016), and ethylene (Yu et al., 2013). The transcript accumulation of HY5 in cells light-mediated signal transduction through phytochromes and cryptochromes (Osterlund et al., 2000; Wang et al., 2001).

## Light-Mediated Regulation of Root Development

Auxin the most important phytohormone has been known to play a pivotal role in regulating root growth and development by virtue of its polar transport ability. PIN transporters have been reported to control to control the localization of auxin to the plasma membrane and hence their polar transport ability. E3 ubiquitin ligase, *CONSTITUTIVE PHOTOMORPHOGENIC 1* (*COP1*) has been reported to negatively regulate the light-induced transport of auxin to the roots by inhibiting the proteins involved in the light response. Likewise, COP1 has been identified as a repressor of PIN1 and hence HY5 thereby inhibiting the auxin transport to the roots (Kiss et al., 2003; Sheerin et al., 2015; Rakusová et al., 2016; Lee et al., 2017). Analysis of phyB mutants of *Lotus japonicus* has shown impaired nodule formation due to downregulation of JA responsive genes. A crucial observation in this perspective was the involvement of shoot phyB in enhancing the JA mediated signaling directed to root photomorphogenesis (Suzuki et al., 2011). Accumulation of photosynthetic products is also an essential factor regulating the circadian clock in roots and hence the emergence of lateral roots. A hierarchical multi-oscillator network driven by the shoot circadian clock modulates the circadian rhythm in roots. These observations clearly suggest the functional involvement of the photosynthetic signals in controlling the clock functions in the roots (Takahashi et al., 2015; Voß et al., 2015). Activation of NITRATE TRANSPORTER 2.1 by a HY5 transcription factor in shoots facilitates carbon assimilation and nitrogen uptake in the roots and thereby maintaining the nutrient balance. Researches carried out with *Cyclophilin 1* (*Cyp1*) encoding a peptidyl-prolyl cis/trans isomerase mutant in tomato have shown impairment of lateral and secondary root growth. Light induces the translocation of Cyp1 from phloem to the roots and thereby clearly indicating that it plays an essential role in above ground perception of light and hence auxin-mediated photomorphogenesis of roots (Spiegelman et al., 2015). The role of phytochrome chromophore or phytochrome themselves in relation to photoregulation of root elongation and sensitivity to JA was further elucidated by analysis of PFB-deficient elongated hypocotyl2 (hy2-1) mutant or *BILIVERDIN REDUCTASE* (*BVR*) overexpressor in *Arabidopsis* (Costigan et al., 2011). Light has been shown to play a role in gravitropism that is the movement of plants toward gravity and roots are the positive proponents of the same with root cap suggested to be the sensor. However, the mechanism governing light-mediated regulation of gravitropism is still unknown. In a recent study conducted with maize roots treated with auxin, synthesis inhibitors showed inhibition of root curvature and no increase in IAA content and light irradiation of roots with white light showed increased transcript accumulation of *Zmyuc* genes in the root apex. Based on the observations it was suggested that light-induced accumulation and spatial distribution of IAA and hence the gravitropic U-turn of the roots (Suzuki et al., 2016). Expressions of cryptochromes have been detected in *Arabidopsis* roots both at transcription and post-transcriptional levels and hence have been suggested to affect root elongation via indirect pathways. Recent researches have also identified more direct evidence of CRYs on root growth and development. Mutant analysis of *cry1* and *cry2* have shown inhibition of root growth, reduced levels of free auxin and increased accumulation of flavonoids. In addition, transcription factors like HY5, *GOLDEN2-LIKE2* (*GLK2*) are modified by light signals transduced by the auxin/cytokinin signal transduction cascade and hence initiating the greening of the roots (Kobayashi et al., 2012; Mo et al., 2015).

## Phytohormone Interaction for Gynoecium Development and Fruit Morphogenesis

The gynoecium is composed of carpels which are the female reproductive organs that surround the ovules in the flowering plants. It may consist of one or more uni-carpellate pistils. At the base of the carpel is the ovary which can develop multiple multicellular structures known as the ovules. Gynoecium development is extremely important and very challenging in research for the identification of angiosperms. Auxin is playing a very crucial role in plant reproduction as it regulates the development of both male and female reproductive organs. Another response of auxin is its role in development and growth of fruits. For most plants, the plant hormone auxin has been shown to play a crucial role for gynoecium and ovule development (Pagnussat et al., 2009; Larsson et al., 2014; Panoli et al., 2015). Recent studies from Figueiredo et al. (2016), have also shown that auxin produced by the endosperm is vital for seed coat differentiation and fruit growth without fertilization. More recently, Larsson et al. (2017) have detected low auxin sensor in preanthesis ovules. Anther- dependent signals repress auxin export from the ovule during the process of anthesis, through the immature phloem in the funiculus. It’s a process that can ensure successful coordination of the self-fertilization process. The accumulation of auxin inside the ovule after the fertilization can stimulate auxin responses to coordinate embryo, seed and fruit development. Though, it is remaining elusive the origin of the auxin transported to the apical cell and auxin’s role for zygote elongation. The group (Larsson et al., 2017) has demonstrated that the egg cell detects auxin before even fertilization. Two auxin biosynthetic genes (*YUC2* and *YUC5*) have been observed to be expressed in the central cell before fertilization. The auxin sensing from the FG6 stage might result from auxin influx from both the central cell and the integuments.

Two groups (Moubayidin and Ostergaard, 2014; Larsson et al., 2017) have shown the expression patterns of different PIN auxin transporters (PIN1, PIN3, and PIN7) during gynoecium development, focusing on the importance of PIN1. The transitions between ovule and seed as well as between gynoecium and fruit are crucial for plant survival. During anthesis, PIN1 becomes more internalized throughout the vasculature, while gynoecium shows a delay development in order to give to the anthers of the stamens the time to contact the stigma for successful fertilization. Thus PIN1 localization and DR5 reporter expression data suggest that the auxin response in medial and lateral domains is different from the early developmental stage (Larsson et al., 2017). According to this group, there is a difference in timing of the development of the vasculature in the medial and lateral domains using as a marker the *Aux2*: GFP.

The cytokinin (CK) is known to play an important role in the regulation and in the development of the shoot and root apical meristems (Su et al., 2011; El-Showk et al., 2013). It has been observed a reduce ovule formation when cytokinin biosynthesis in impaired (Werner et al., 2003; Higuchi et al., 2004). Recent studies have shown that a treatment with exogenous cytokinin at developing flowers, resulted not only to cause proliferation of medial domain structures but also in the modification of the apical-basal within the gynoecium (Zuniga-Mayo et al., 2014). A cross-talking signaling pathway between the two plant hormones, auxin and cytokinin plays a vital role at the development of the shoot and root apical meristem (Su et al., 2011; El-Showk et al., 2013). Through *N*-naphtylphthalamic acid (NPA) treatment the observed apical-basal defects have been characterized by a reduced number of valves resulting in blocking of auxin transport, suggesting that there is an interplay in signaling pathways between auxin and cytokinin. Cytokinins promote the cell proliferation process at shoot apical meristems (Leibfried et al., 2005). Marsch-Martínez et al. (2012) have used a transactivation system to drive isopentenyl-transferase 7 (IPT7) or cytokinin oxidase 3 (CKX3) expression from fruit promoters and they have observed a decrease on cytokinin levels at the external medial tissue. CKX are the enzymes being responsible for CK breakdown, and mutations in some of these enzymes results in increased seed yield (Ashikari et al., 2005; Bartrina et al., 2011). Some groups have used synthetic reporters such as DR5 and TCS in order to be able to visualize the auxin and cytokinin responses in the gynoecium (Ostergaard, 2009; Marsch-Martínez et al., 2012). They have shown that CK and auxin responses in the gynoecium have complementary expression patterns and have suggested an antagonistic relationship between CK and auxin.

### Flowering Time

Flowering time is an important aspect that directs reproductive process in plants. Photoperiod or length of the daylight influences the timing of flowering. Plants are categorized based on the time of flowering (i) long day plants are those that flower when the length of the daylight is less than the critical length, (ii) short day plants flower when they have more daylight than the critical length and (iii) day neutral plants flower independently of day length. In *Arabidopsis* and crop plants *phyA, phyB*, and *cry2* were the first photoreceptors to be reported to regulate flowering time. Mutations in *PHYB* or *PHYC* in rice causes a slight alteration in the flowering but a simultaneous mutation in *PHYA*, as well as *PHYB* or *PHYC*, results in early flowering (Takano et al., 2005). Similar results have been observed for both *PHYC* and *PHYB* null mutants in wheat (Chen et al., 2014; Pearce et al., 2016). Transcriptome analysis of the aforementioned mutants also showed that *PHYB* also plays an essential role in the biosynthesis of phytohormones like BR, auxin, ABA and ethylene which are suggested to be involved in the photoperiodic regulation of flowering (Pearce et al., 2016). The control exerted in regulating the endogenous levels of GA is the most important for light-induced flowering. The downregulation of *GA20OX* controlling GA biosynthesis resulted in delayed flowering in FR enriched environment indicating its participation in phytochrome-dependent flowering (Hisamatsu and King, 2008). Recent studies conducted with photomorphogenic mutant or transgenic lines of rice, tomato, and with *Arabidopsis* have provided valuable information regarding the functional redundancy and interaction of different photoreceptors participating in several signaling pathways contributing to plant growth and development. Researches over the years have identified the role of light in seed germination by modulating the ABA and GA signaling pathway through the interaction between phytochromes and PIF1 (de Wit et al., 2016). PIFS have also been hypothesized to be an important contributor in the transition of seedlings to photomorphogenic from skotomorphogenic mode by modulating the levels of GA (de Wit et al., 2016). Tomato polycotyledon mutant (*pct1-2*) showed sequential and increased flowering as well as enhanced auxin transport in comparison to wild-type which blooms at the same time as the mutant (Al-Hammadi et al., 2003; Kharshiing et al., 2010a,b). The flowers in the mutants (*pct1-2*) exhibited male sterility due to lack of anther dehiscence. The auxin transporters *ABCB1* and *ABCB19* were reported to be involved in regulating anther dehiscence in *Arabidopsis* (Cecchetti et al., 2008, 2015). The identification of *ABCB19* (ATP-Binding Cassette B19) in *Arabidopsis* as a substrate for *Phot1* (Phototropin1) justifies the overlap of hormone signaling and the photoreceptor in plants (Christie et al., 2011). Moreover, phytohormones have been reported to regulate development and ripening of fruit in tomato (Gupta et al., 2014). Analysis of tomato *sh* (short root) mutant has shown the increased production of nitric oxide, the second messenger of signaling pathways for several plant hormones subsequently resulted in inhibition of fruit growth and ripening suggesting the possibility of hormonal cross-talk (Negi et al., 2010; Bodanapu et al., 2016). Under the present stage of global climate change, these different photoreceptor interactions become very relevant. In future, researches focused on the functional characterization of the interaction of different photoreceptors will help in contributing to improved yield and productivity of crop plants for both and non-food use.

## Conclusion and Remarks

Plant growth and development is depending on phytohormone biosynthesis and its transport to plant tissues that necessitate phytohormones for growth. The effect of a hormone is well established by different pathways that are organized and connected through a complex network of feedback regulations. The processes of seed germination and seed dormancy are influenced by plant hormones. Both these processes can affect crop production and the phytohormones which are produced by synergistic interactions between plants and soil bacteria can affect seed germination. Some plant genes, which are necessary for the activity of plant hormones and the other plant genes, are activated by plant hormones. The linkage of MYB96 with clock components possibly shapes the circadian gating of ABA responses. The circadian clock defines the diurnal ABA induction of MYB96 and its downstream genes that peak around dusk. Accordingly, misregulation of TOC1 leads to the disruption of ABA-mediated induction of MYB96 and MYB96-mediated ABA signaling. ABA is a crucial player among phytohormones that affect the developmental and physiological events in plants. A number of studies have demonstrated the importance that ABA plays in inhibition of germination process of seed growth, limiting the responses during abiotic stresses, and its role in the important process of signaling during phosphorylation. It has also been demonstrated that ABA inhibits shoot growth in watered plants. ABA deficiency in drought-stressed plants can cause enhancement of shoot growth which can be confirmed with the endogenous ABA accumulation being responsible for plant growth inhibition. Flower development is an essential process for sexual reproduction. As the critical female reproductive structure in plants, the gynoecium plays a vital role for the agricultural productivity. Highlights of this process are the important role of auxin in the differential development and in the termination of the floral meristem, and also the signaling interaction between auxin and cytokinin during gynoecial development. Cytokinin signaling plays a crucial role in the auxin biosynthesis regulation and transport in the ovary position of the gynoecium. Communication between cytokinin-auxin signaling pathways has to be the subject of a continues research.

Future studies will aim to tighten up the loose ends in phytochrome and phytohormone signaling and elucidate how these signalings intersect with various signaling pathways to coordinately regulate the diverse physiological and developmental processes. Further genome-genetic studies will be necessary to elucidate the complicated mechanisms by engineering and generate stress tolerant plants in order to locate and unravel the key components that influence the developmental processes mediated by ABA. Furthermore, it becomes essential to elucidate all ABA-responsive genes to gain a clear view of the complex feature of abiotic and biotic stresses.

## Author Contributions

PL was responsible for editing the manuscript, writing the introduction and also writing the phytohormone section. RR and JM were responsible for the abstract and writing the phytochrome section.

## Conflict of Interest Statement

The authors declare that the research was conducted in the absence of any commercial or financial relationships that could be construed as a potential conflict of interest.
